# Anti-protein phosphatase magnesium-dependent 1A-IgM levels in patients with active ankylosing spondylitis: a potential biomarker

**DOI:** 10.1186/s42358-024-00412-9

**Published:** 2024-09-13

**Authors:** Yeo-Jin Lee, Eun-Ju Lee, Soo Min Ahn, Seokchan Hong, Ji Seon Oh, Chang-Keun Lee, Bin Yoo, Yong-Gil Kim

**Affiliations:** 1 Department of Rheumatology, Asan Medical Center, University of Ulsan College of Medicine, 88, Olympic-ro 43-gil, Songpa-gu, Seoul, 05505 Republic of Korea; 2 Department of Information Medicine, Big Data Research Center, Asan Medical Center, Seoul, Republic of Korea

**Keywords:** Ankylosing spondylitis, Anti-PPM1A, Rheumatoid arthritis, Diagnosis

## Abstract

**Background:**

Ankylosing spondylitis (AS) has been known to have auto-inflammatory nature; hence, the efficacy of autoantibodies is low. However, studies on autoantibodies are ongoing, with some studies showing associations. Previous studies showed that anti-protein phosphatase magnesium-dependent 1A (PPM1A) IgG was increased in patients with AS and associated with radiographic progression. However, the diagnostic usefulness was limited due to relatively low sensitivity and specificity. This pilot study evaluated the diagnostic utility of anti-PPM1A-IgM and anti-PPM1A-IgG in patients with active AS.

**Methods:**

Serum samples were obtained from the registry cohort of a single tertiary center in Korea. Serum levels of anti-PPM1A-IgG/IgM were measured by direct ELISA. Receiver operating characteristic (ROC) analysis was used to predict the diagnostic sensitivity and specificity of serum anti-PPM1A-IgG/IgM.

**Results:**

Samples were collected from 28 patients with active AS, 16 healthy controls (HCs), and 28 patients with rheumatoid arthritis (RA). Although total serum IgM was lower in the RA and AS groups than in the HC group, anti-PPM1A-IgM was significantly lower in the AS group than in the other groups. In evaluating the diagnostic utility of anti-PPM1A-IgG/IgM for AS patients compared with HCs, the area under the curve (AUC) of anti-PPM1A-IgM was 0.998 (sensitivity 96.4%, specificity 100.0%). When ROC analysis of anti-PPM1A-IgM for AS patients compared with RA patients was conducted, sensitivity was 78.6% and specificity was 71.4%, with an AUC of 0.839.

**Conclusion:**

Decreased anti-PPM1A-IgM levels in AS patients suggests a potential role for anti-PPM1A-IgM in the diagnosis of active AS.

## Introduction

Ankylosing spondylitis (AS) is a chronic disease that debilitates the working-age population, and active disease status is associated with functional impairment, ankylosis, and reduction of bone mass [1, 2]. With the advent of biologics, studies have reported the importance of early treatment initiation [3]. Studies evaluating the efficacy of tumor necrosis factor (TNF) inhibitors in AS have identified decreased inflammatory markers and improvement of back pain, function, and quality of life in AS patients treated with TNF inhibitors [4–6].

Therefore, identifying and initiating effective treatment is important for patients with active AS. Unlike other rheumatic diseases, especially systemic lupus erythematosus, in which autoantibody positivity is the entry criterion, AS has been known to have auto-inflammatory etiology, so the diagnostic efficacy of autoantibodies is low [7, 8]. However, studies of autoantibodies are ongoing, with some studies identifying associations between autoantibodies and AS [9–11]. For example, protein phosphatase magnesium-dependent 1A (PPM1A) is a serine/threonine protein phosphatase that regulates bone morphogenetic protein (BMP) and Wingless (Wnt) signaling [12–14]. Previous studies showed that anti-PPM1A-IgG was increased in patients with AS and was associated with radiographic progression [15, 16]. However, the diagnostic utility of anti-PPM1A-IgG was limited due to its relatively low sensitivity and specificity [16].

Therefore, the present study aimed to evaluate the utility of anti-PPM1A-IgM, rather than IgG, in patients with active AS prior to biologic agent administration relative to healthy controls and patients with rheumatoid arthritis (RA) as a disease control.

## Materials and methods

### Patients and data collection

Serum samples were obtained from the registry cohort of a single tertiary center in Seoul, Korea (Asan medical center) from March 2015 to March 2016. Enrolled patients were recruited during the same period. All AS patients met 1984 modified New York criteria and BASDAI ≥ 4 and had not received a biologic agent. RA patients that met 2010 ACR/EULAR classification criteria with moderate to high disease activity and had not received a biologic agent were also analyzed. The Disease Activity Score in 28 Joints with Erythrocyte Sedimentation Rate (DAS-28-ESR) index was used to assess disease activity in RA. Sera from patients and healthy controls were obtained with the approval of the Asan Medical Center Institutional Review Board (2014-0237, 2015-0274). Informed written consent was obtained from all participants. This study was performed in accordance with the Declaration of Helsinki and its later amendments. Electronic medical records, including white blood cell count (WBC), erythrocyte sedimentation rate (ESR), C-reactive protein (CRP), medications, Bath AS Disease Activity Index (BASDAI), and radiographic findings, were obtained. Authors had no access to information that could identify individual participants during or after data collection.

### Measurement of anti-PPM1A antibody levels

Serum levels of anti-PPM1A-IgG/IgM were measured by direct ELISA, and expressed as optical density (O.D.) [15]. The levels of serum anti-PPM1A antibodies were quantified as follows. Nunc‐Immuno‐Maxisorp 96‐well plates were coated with 100 µL/well of PPM1A (1 µg/mL) (YbdY, Seoul, Korea) in PBS at 4 °C overnight [15]. Plates were washed with PBS‐Tween 20 (PBST; 0.05%, v/v), and subsequently blocked with 2% Chon Block (Chondrex Inc., Redmond, WA, USA) in 0.05% PBST for 1 h at room temperature. After washing, 100 µL serum diluted 1:50 with 2% Chon Block in 0.05% PBST was added and incubated for 1 h at room temperature. After washing, peroxidase‐conjugated AffiniPure donkey anti-human IgM or peroxidase‐conjugated AffiniPure donkey anti-human IgG (Jackson Immuno Research Inc., West Grove, PA, USA) was added and incubated for 1 h at room temperature. Reactions were developed with 100 µL 3,3′,5,5′‐tetramethylbenzidine (TMB) substrate for 15 min at room temperature. Colorimetric reactions were stopped with 50 µL H_2_SO_4_ and optical density was read at 450 nm.

### Statistical analyses

Continuous variables were represented as median values (interquartile ranges [IQR]) or mean values (standard deviation [SD]), and categorical variables were represented as frequencies and percentages (n, %). To compare parametric data, a t-test was used, and for nonparametric data, a Mann-Whitney U-test and a Kruskal-Wallis test were used. Categorical variables were compared using the Chi-squared test and Fisher’s exact test. For comparing three groups, Analysis of Variance (ANOVA) and post-hoc Bonferroni analysis were used. Logistic regression analysis was performed to identify factors associated with AS patients compared with RA. Receiver operating characteristic (ROC) analysis was used to predict diagnostic sensitivity and specificity. Analysis requiring laboratory data was conducted except for one patient’s missing data. A *p*-value < 0.05 was considered significant, and all statistics were performed using R, version 1.4.1717, or SPSS software, version 21.0 (IBM, Armonk, NY).

## Results

### Baseline characteristics of AS and RA patients before treatment with biologics

A total of 72 participants were enrolled in the present study, and samples were collected from 16 HCs, 28 patients with AS, and 28 patients with RA. Baseline characteristics of AS and RA patients are shown in Table 1. The mean age (years) at enrollment was 33.5 (±11.9) for AS patients, 51.8 (±13.6) for RA patients, and 35.0 (±9.4) for HCs. Twenty-five patients (89.3%) in the AS group, six patients (20%) in the RA group, and seven HCs (43.8%) were male. The disease duration of AS patients from diagnosis to study enrollment was a median of 8 months (IQR: 3.3–26.8 months).

**Table 1:** Baseline characteristics of AS patients prior to treatment with biologics, compared to RA patients and healthy controls

	AS (*n* = 28)	RA (*n* = 28)	*p*-value*	HC (*n* = 16)	*p*-value**
Age, years	33.5 (±11.9)	51.8 (±13.6)	<0.001	35.0 (±9.4)	0.717
Male	25 (89.3)	6 (20)	<0.001	7 (43.8)	0.001
WBC, x10^3^/uL	7407.1 (±1447.1)	7589.3 (±2601.8)	0.747		
ESR, mm/hr	25.5 (21.3–56.0)	25.0 (14.8–50.3)	0.385		
CRP, mg/dL	1.25 (0.39–2.14)	0.13 (0.10–0.76)	<0.001		
NSAID use			0.042		
None	0 (0)	5 (17.9)			
As needed	1 (3.6)	0 (0)			
Regular use	27 (96.4)	23 (82.1)			
DMARD use					
Sulfasalazine	26 (92.9)	0 (0)	<0.001		
Methotrexate	3 (10.7)	26 (86.7)	<0.001		
Hydroxychloroquine	0 (0)	17 (56.7)	<0.001		
Leflunomide	0 (0)	1 (3.3)	>0.999		
Use of corticosteroid	5 (17.9)	24 (85.7)	<0.001		
BASDAI	6.00 (5.35–6.80)				
Acute anterior uveitis	6 (21.4)				
HLA-B27	27 (96.4)				
DAS-28-ESR		4.9 (±1.3)			

Values are presented as mean (standard deviation), median (interquartile range) or n (%)

*AS* ankylosing spondylitis, *RA* rheumatoid arthritis, *HC* healthy control, *WBC* white blood cell count, *ESR* erythrocyte sedimentation rate, *CRP* C-reactive protein, *NSAID* nonsteroidal anti-inflammatory drug, *DMARD* disease modifying anti-rheumatic drug, *BASDAI* Bath Ankylosing Spondylitis Disease Activity Index, *HLA-B27* human leukocyte antigen-B27, *DAS-28-ESR* disease activity score-28-erythrocyte sedimentation rate

*p*-values are indicated as follows: * for AS vs. RA, ** for AS vs. HC

Baseline laboratory data were available in all 28 AS patients and 27 RA patients. Baseline WBC and ESR did not significantly differ between groups, but baseline CRP was significantly higher in AS patients relative to RA patients (1.25 [IQR, 0.39–2.14] vs. 0.13 [IQR, 0.10–0.76], *p* < 0.001). Among 28 patients with AS, HLA-B27 positivity was present in 27 patients (96.4%) and median BASDAI was 6.00 (5.35–6.80).

### Anti-PPM1A levels in HCs and patients with AS or RA

**Fig. 1:**
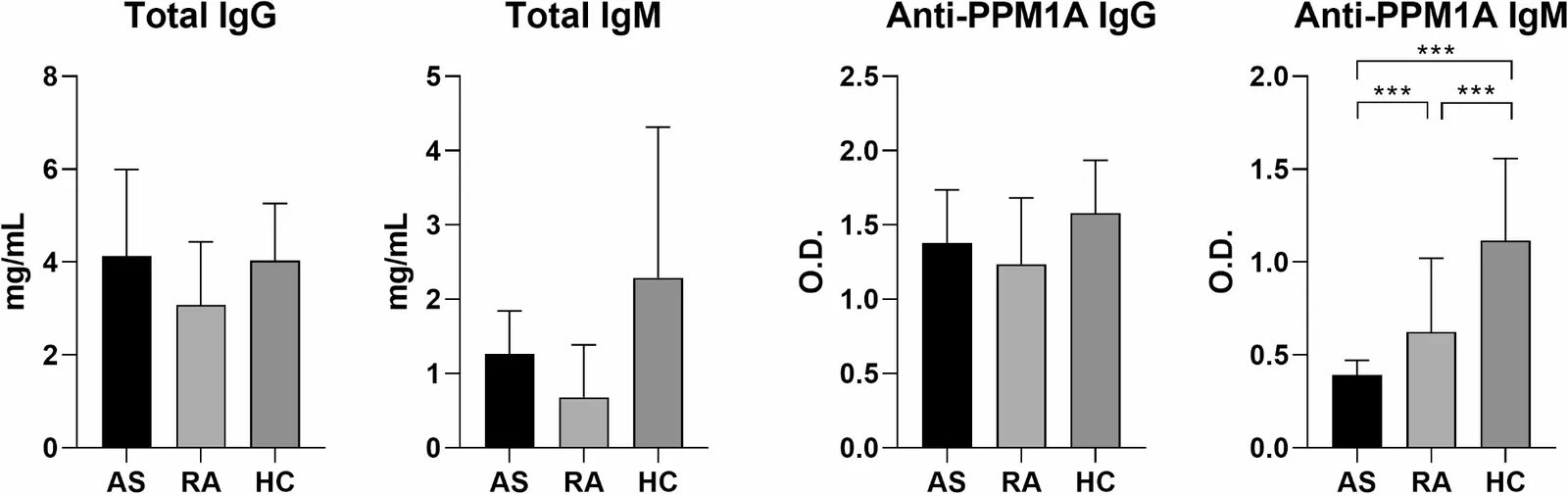
Immunoglobulin levels of healthy control subjects and patients with AS or RA. Values are median (interquartile range); *HC* healthy control, *AS* ankylosing spondylitis, *RA* rheumatoid arthritis, *PPM1A* protein phosphatase magnesium dependent 1A, *O.D.* optical density. ***p < 0.001

Anti-PPM1A-IgM was measured in a total of 72 patients (Fig. 1). Total IgM levels were lower in AS and RA patients compared to HCs, though the differences were not statistically significant. However, the anti-PPM1A-IgM level (O.D.) was significantly lower in the AS group than those in the other two groups {(0.39 in AS vs. 0.62 in RA (*p* < 0.001) and 1.12 in HC (*p* < 0.001)}. Total IgG and anti-PPM1A-IgG levels did not significantly differ between HCs, AS patients, and RA patients.

### Diagnostic utility of anti-PPM1A-IgM level in AS

**Fig. 2:**
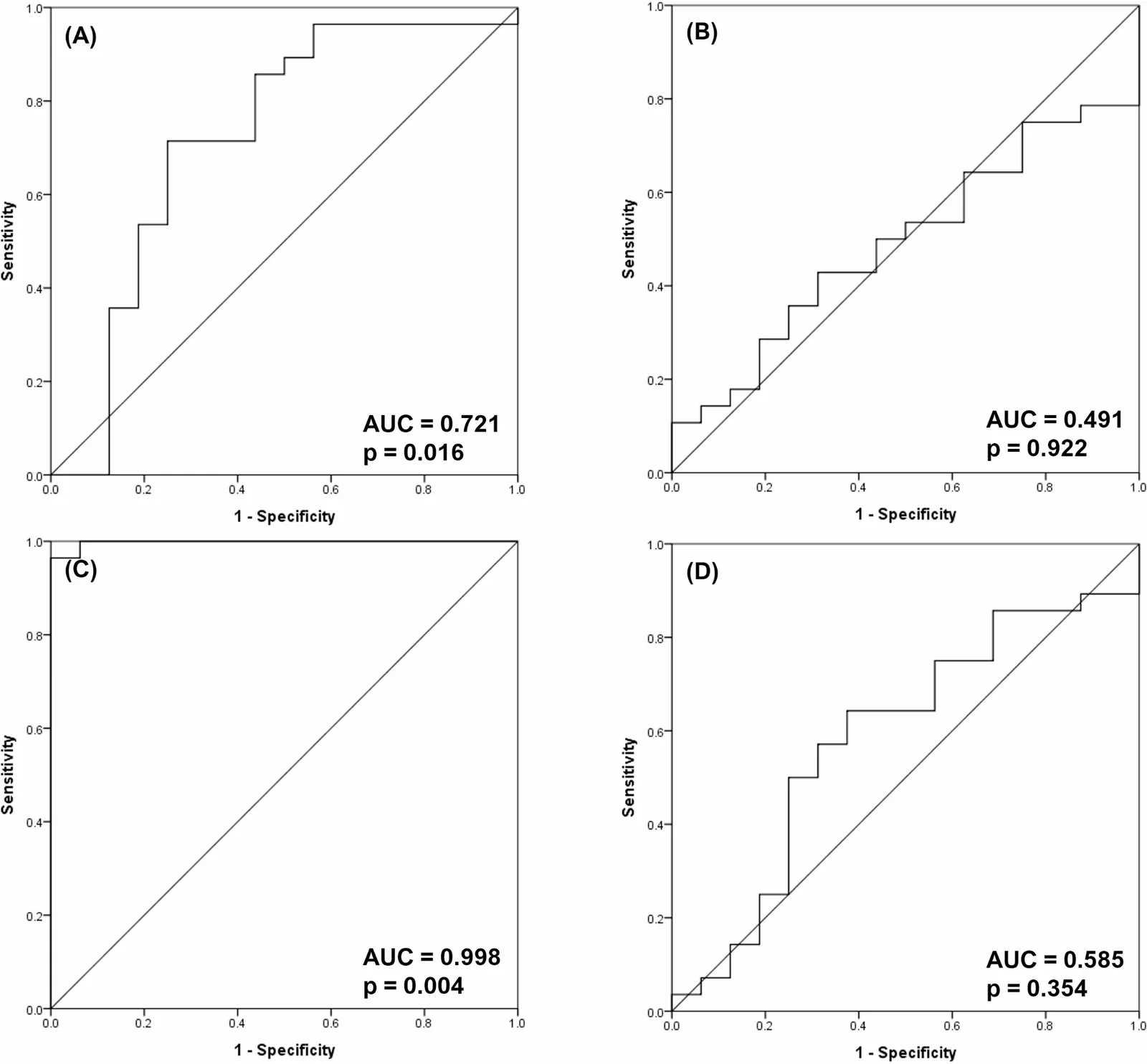
ROC analysis predicting diagnostic utility of AS patients compared with HCs. **A**) Area under the curve (AUC) of total IgM predicting diagnostic utility of AS patients, **B**) AUC of total IgG, **C**) AUC of anti-protein phosphatase magnesium dependent 1A (PPM1A)-IgM, **D**) AUC of anti-PPM1A-IgG

**Fig. 3:**
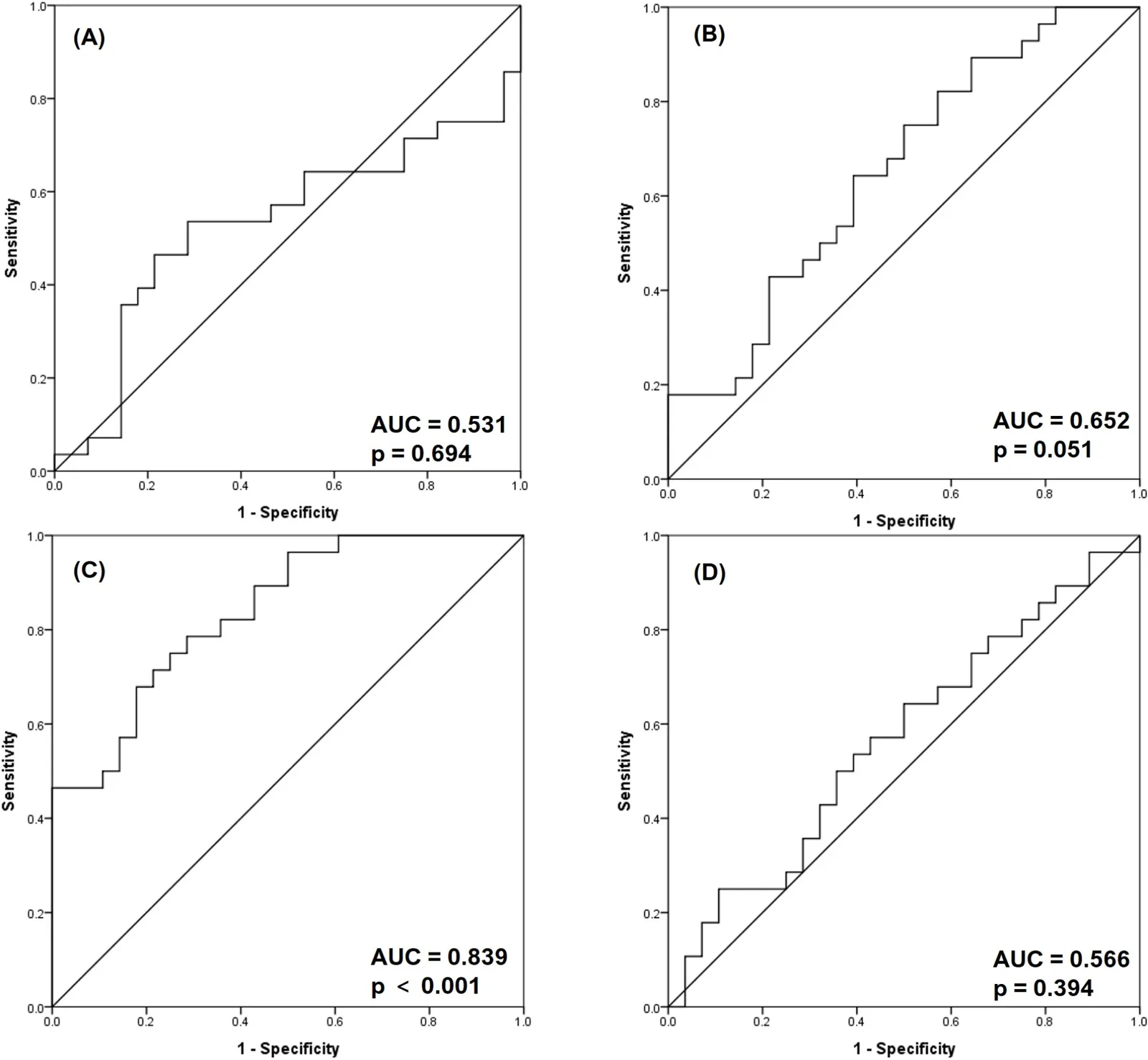
ROC analysis predicting diagnostic utility of AS patients compared with RA patients. **A**) Area under the curve (AUC) of total IgM predicting diagnostic utility of AS patients, **B**) AUC of total IgG, **C**) AUC of anti-protein phosphatase magnesium dependent 1A (PPM1A)-IgM, **D**) AUC of anti-PPM1A-IgG

In consideration of differences in baseline characteristics of patients with AS and HCs, adjusted logistic regression analysis was performed (Table 2). After adjusting for sex, anti-PPM1A-IgM showed a trend of association with AS (OR 0.146, *p* = 0.067). To identify the diagnostic utility of variables in patients with AS compared with that of HCs, ROC analysis was performed. AUC of anti-PPM1A-IgM was analyzed, with a value of 0.998 (*p* = 0.004, sensitivity 96.4%, specificity 100.0%) (Fig. 2). After adjusting for age and sex, anti-PPM1A-IgM showed significant association with AS patients compared with RA patients (OR 0.582, *p* = 0.046) (Table 3). When ROC analysis was performed to compare the diagnostic utility of anti-PPM1A-IgM in AS patients with that of RA patients, AUC value was 0.839 (*p* < 0.001, sensitivity 78.6%, specificity 71.4%) (Fig. 3).

**Table 2:** Unadjusted and adjusted analysis of factors associated with AS patients compared with HC

	Unadjusted OR (95% CI)	*P*-value	Adjusted OR* (95% CI)	*P*-value
Age	0.988 (0.928–1.052)	0.709		
Male	10.714 (2.269–50.598)	0.003		
Total IgM, mg/mL	0.965 (0.928–1.002)	0.066	0.969 (0.932–1.008)	0.118
Total IgG, mg/mL	1.002 (0.974–1.031)	0.890	1.017 (0.979–1.056)	0.381
Anti-PPM1A-IgM (O.D)	0.125 (0.015–1.055)	0.056	0.146 (0.019–1.143)	0.067
Anti-PPM1A-IgG (O.D)	0.936 (0.813–1.078)	0.359	0.897 (0.759–1.061)	0.206

*OR* odds ratio, *CI* confidence interval, *HC* healthy control, *AS* ankylosing spondylitis, *PPM1A* protein phosphatase magnesium dependent 1A

*Adjusted for sex

**Table 3:** Unadjusted and adjusted analysis of factors associated with AS patients compared with RA patients

	Unadjusted OR (95% CI)	*P*-value	Adjusted OR* (95% CI)	*P*-value
Age	0.892 (0.840–0.948)	<0.001		
Male	30.556 (6.820–136.895)	<0.001		
Total IgM, mg/mL	1.004 (0.976–1.033)	0.785	1.001 (0.963–1.041)	0.956
Total IgG, mg/mL	1.026 (0.999–1.053)	0.055	1.059 (0.993–1.131)	0.083
Anti-PPM1A-IgM (O.D)	0.492 (0.322–0.752)	0.001	0.582 (0.343–0.990)	0.046
Anti-PPM1A-IgG (O.D)	1.056 (0.932–1.195)	0.393	1.001 (0.818–1.224)	0.995
CRP, mg/dL	2.280 (1.080–4.815)	0.031	1.837 (0.803–4.202)	0.150
ESR, mm/hr	1.007 (0.988–1.027)	0.462	1.019 (0.984–1.056)	0.298

*OR* odds ratio, *CI* confidence interval, *AS* ankylosing spondylitis, *RA* rheumatoid arthritis, *PPM1A* protein phosphatase magnesium dependent 1A, *CRP* C-reactive protein, *ESR* erythrocyte sedimentation rate

*Adjusted for age, sex

## Discussion

The present study demonstrated that anti-PPM1A-IgM is useful for diagnosis of active AS. The use anti-PPM1A-IgM levels demonstrated diagnostic power with a specificity of 100% for diagnosing AS compared to healthy controls.

Previous studies of PPM1A autoantibodies have focused on anti-PPM1A-IgG levels, which were significantly associated with radiographic severity and disease activity in AS patients [15, 16] Also, higher levels of anti-PPM1A-IgG suggested a high probability of radiographic progression [15] However, the utility of anti-PPM1A autoantibodies in AS diagnosis has not been previously evaluated. In the present study, the AUC of anti-PPM1A-IgG for AS patients was only 0.585 when compared with healthy controls and was not statistically significant. However, the AUC of anti-PPM1A-IgM was 0.998 (*p* = 0.004). Therefore, anti-PPM1A-IgM is predicted to be important for diagnosis of active AS. However, in this study, we were unable to provide an exact explanation for the mechanism behind the increase in anti-PPM1A-IgG levels and the decrease in anti-PPM1A-IgM levels in patients with AS. While elevated IgG autoantibodies and decreased IgM autoantibodies in patients with coronary artery disease have been demonstrated in other fields, the specific reasons were not clearly elucidated in that study either [17, 18]. The proposed hypotheses in that study include an increase in IgM consumption, enhanced clearance or uptake from vessel walls, or genetic differences in IgM autoantibody levels [18]. Additional studies are needed to elucidate and explain these findings.

Although there is no confirmatory blood test for diagnosis of AS, several studies have reported potential biomarkers for AS. HLA-B27 positivity is most commonly considered at diagnosis, but its positivity rate varies by geographical region and ethnicity [19–21]. In some tribal and native populations, the HLA-B27 positivity rate was ranged from 30 to 50%, compared with 8% for Western Europeans [19–22]. A meta-analysis demonstrated that polymorphisms of endoplasmic reticulum aminopeptidase 1 (ERAP1), together with HLA-B27, were associated with AS in European patients [23]. However, another study reported that ERAP1 is useful only in patients with HLA-B27 positivity [24]. Vascular endothelial growth factor (VEGF) also had been evaluated as a biomarker for AS [25–27]. Studies of VEGF have primarily evaluated the association with disease activity or radiographic progression. However, there was no significant difference in the presence of VEGF polymorphisms between HCs and AS patients, and there was no significant difference in VEGF levels between RA and AS patients [25, 26]. Therefore, the findings of the present study are noteworthy, in that it reported significant utility of a biomarker for diagnosis of active AS.

This study has several limitations. First, due to the nature of the pilot trial, the number of patients enrolled in the study is small, and patients were enrolled from a single center. Additionally, no further laboratory data were available for 16 HCs, so comparison of some laboratory data, including CRP, between HCs and AS patients was not possible. Validation in additional patient cohorts is required to confirm the findings of the study. Additionally, while we selected RA patients as a disease control, we acknowledge that there are intrinsic differences in disease characteristics. Despite adjusting for age and gender in our analysis, these differences may necessitate careful interpretation of the results. Finally, as this was the first study focusing on the role of anti-PPM1A-IgM in patients with active AS, further studies are needed to explore its relevance in inactive AS and axial spondyloarthritis (axSpA).

In conclusion, we observed the decrease in anti-PPM1A-IgM levels in AS patients suggests a potential role for anti-PPM1A-IgM in the diagnosis of active AS. While these findings are promising, given the small sample size and limited data, further research is needed to fully establish its role. Nonetheless, anti-PPM1A-IgM measurements could be considered as a supplementary diagnostic tool for active AS, particularly in situations where radiographic evaluation of sacroiliitis is not feasible.

## Data Availability

The data that support the findings of this study are available from the corresponding author, YG Kim, upon reasonable request. The data are not publicly available due to ethical review board restrictions as it contains information that could compromise the privacy of research participants.
